# Artificial Intelligence in Acute Ischemic Stroke Subtypes According to Toast Classification: A Comprehensive Narrative Review

**DOI:** 10.3390/biomedicines11041138

**Published:** 2023-04-10

**Authors:** Giuseppe Miceli, Maria Grazia Basso, Giuliana Rizzo, Chiara Pintus, Elena Cocciola, Andrea Roberta Pennacchio, Antonino Tuttolomondo

**Affiliations:** 1 Department of Health Promotion, Mother and Child Care, Internal Medicine and Medical Specialties (ProMISE), Università Degli Studi di Palermo, Piazza Delle Cliniche 2, 90127 Palermo, Italy; 2 Internal Medicine and Stroke Care Ward, University Hospital, Policlinico “P. Giaccone”, 90141 Palermo, Italy

**Keywords:** artificial intelligence, ischemic stroke, machine learning, deep learning, toast classification

## Abstract

The correct recognition of the etiology of ischemic stroke (IS) allows tempestive interventions in therapy with the aim of treating the cause and preventing a new cerebral ischemic event. Nevertheless, the identification of the cause is often challenging and is based on clinical features and data obtained by imaging techniques and other diagnostic exams. TOAST classification system describes the different etiologies of ischemic stroke and includes five subtypes: LAAS (large-artery atherosclerosis), CEI (cardio embolism), SVD (small vessel disease), ODE (stroke of other determined etiology), and UDE (stroke of undetermined etiology). AI models, providing computational methodologies for quantitative and objective evaluations, seem to increase the sensitivity of main IS causes, such as tomographic diagnosis of carotid stenosis, electrocardiographic recognition of atrial fibrillation, and identification of small vessel disease in magnetic resonance images. The aim of this review is to provide overall knowledge about the most effective AI models used in the differential diagnosis of ischemic stroke etiology according to the TOAST classification. According to our results, AI has proven to be a useful tool for identifying predictive factors capable of subtyping acute stroke patients in large heterogeneous populations and, in particular, clarifying the etiology of UDE IS especially detecting cardioembolic sources.

## 1. Introduction

Stroke is one of the main causes of morbidity and mortality worldwide. According to the World Stroke Organization, there are over 12.2 million new strokes each year. Globally, one in four people over age 25 will have a stroke in their lifetime [1]. Ischemic stroke (IS) is the most frequent kind of stroke (80% of all cases). The correct recognition of the etiology of IS allows tempestive interventions in therapy with the aim of treating the cause and preventing a new cerebral ischemic event. Nevertheless, the identification of the cause is often challenging and is based on clinical features and data obtained by imaging techniques and other diagnostic exams.

Trial of Org 10172 in Acute Stroke Treatment (TOAST) classification system describes the different etiologies of ischemic stroke and includes five subtypes: LAAS (large-artery atherosclerosis), CEI (cardio embolism), SVD (small vessel disease), ODE (stroke of other determined etiology) and UDE (stroke of undetermined etiology) [2].

Time of diagnosis is crucial for time-related treatments, which allow the improvement of clinical outcomes and the reduction of disability, according to the adage, “time is brain” [3]. In clinical practice, early identification of the mechanism of acute ischemic stroke is highly dependent on a reliable imaging examination that must be interpreted promptly [4]. Imaging techniques are the cornerstone of the work-up in stroke patients. The commonly used imaging studies are computed tomographic angiography (CTA), magnetic resonance imaging (MRI), and ultrasound (US). Artificial intelligence (AI) is a branch of computer science that experienced huge developments in the past five years, with significant implications for medical imaging. It represents a new technology able to analyze complex data using automated algorithms for obtaining a final output. IS is one of the medical fields that have been extensively affected by the AI revolution [4]. In fact, AI algorithms have been shown to be able to perform accurate lesion classification, detection, and segmentation in brain tissue. AI also has been used for imaging-guided decision-making and outcome prediction. AI models may detect precociously and quantify intracranial hemorrhage, microbleeds, and acute ischemic stroke, which includes the presence of cerebral infarction and large vessel occlusions not always detectable to the human eye [5].

Since the time interval between the onset of IS and its diagnosis and treatment is crucial for a favorable clinical outcome, the rapid solutions offered by the AI model represent a potential tool for the correct and efficient diagnostic classification of ischemic stroke [6]. The aim of this review is to provide overall knowledge about the most effective AI models used in the differential diagnosis of ischemic stroke etiology according to the TOAST classification.

## 2. Overview of Artificial Intelligence

AI can be identified as the use of any device to mimic the human cognitive process and involves learning, applying, and solving complex problems [7]. This became the basis of computer development programmed to “think and reason”. The first AI-based technology was approved in April 2018 by the US Food and Drug Administration, which was an ophthalmic application for screening diabetic retinopathy [8]. After this approval, a growing number of applications followed with different health claims. AI-based methods are increasingly taking up space in the healthcare industry as they have helped to calculate risk, guide treatment, and predict outcomes using advanced algorithms applied to a large multimodality dataset. AI comprehends several models and methods, including machine learning (ML), deep learning (DL), and convolutional neural networks (CNNs). In ML, multiple learning methods are used, each of them with a different task and with the ability to solve different classes of problems ranging from automation of processes to predictive analytics. ML represents a subfield of AI that focuses on developing algorithms that can learn from data and make predictions or decisions based on that learning without being explicitly programmed. The process of learning in ML usually involves training, testing, and validation processes, and the algorithms are categorized into three types: supervised, unsupervised, and reinforcement learning. Supervised learning is the most commonly used method in diagnostic imaging. It includes learning from labeled data, where the algorithm is trained on a set of input-output pairs (i.e., the features and their corresponding labels) and then uses that learning to predict the labels of new, unseen data. Supervised learning is used when the task can be precisely defined, and the algorithm is expected to learn based on the known data. In the medical field, for example, two sets of data can be used, one associated with the chosen outcome and the other one not. Based on the first set of data, the algorithm will learn to assign the correct outcome to the not-labeled group. Unsupervised learning, on the other hand, involves learning from unlabeled data, where the algorithm tries to find patterns or structures in the data without any guidance. In this case, two sets of data should be used, both without the labeled outcome. The algorithm provides the ability to group data into categories based on the similarity of characteristics taken into consideration (laboratory markers, symptoms, age, and gender). Reinforcement learning is a type of learning that involves learning from interactions with an environment, where the algorithm receives rewards or penalties based on its actions and learns to make better decisions over time. Thus, the algorithm completes tasks without previous instructions, but it can learn while failing to complete the task. It is therefore guided with some basic rules in how to perform the task using his own experiences [8,9]. ML can be divided into two types based on whether the features are handcrafted or not. Handcrafted features are usually defined by experts and are believed to be effective in differentiating between different data classes. In contrast, techniques without handcrafted features can learn directly from the data and optimize their problem-solving capabilities without relying on pre-defined features. This approach is often referred to as deep learning, and it has shown remarkable success in various applications, including image recognition, natural language processing, and speech recognition. DP is a subset of machine learning that uses a layered structure of artificial neural networks (Artificial Neuronal Networks, ANNS) inspired by the neural network of the human brain, with the advantage of processing a greater amount of data in different formats such as video, audio, and text [10].

The goal of DL is to simulate the function of the human brain’s neural networks, allowing machines to learn from and make decisions based on raw data without being explicitly programmed to do so. DP is generally based on NN (neural networks) architectures, using multiple layers to gradually extract higher-level features from the inputs. The output from each layer is fed into the next layer, allowing the system to learn increasingly complex representations of the input data. This layered, hierarchical approach allows DL models to learn from vast amounts of data, often achieving state-of-the-art performance in tasks such as image and speech recognition, natural language processing, and more. The success of DL is due in part to the availability of large datasets, powerful computing resources such as graphical processing units, and the development of efficient learning algorithms such as backpropagation. DL has been used to achieve impressive results in a wide range of applications, from self-driving cars and facial recognition to drug discovery and medical diagnosis. The ANN structure can have many layers, and the amounts of layers are proportional to the complexity of the final architecture it can achieve. Some of the most common architectures of DL include convolutional NNs (CNNs), recurrent NNs, variational autoencoders, and generative adversarial NNs [11]. Although this area of learning is much more advanced than the other forms described above, some limitations of the DP must be considered: a large number of training examples are needed to create a more accurate and reproducible model; the number and quality of input data influence the final result, as well the human intervention. In fact, it is up to the physician to interpret whether the characteristics identified by the DL model are compatible with clinical knowledge of the disease and the implications of such findings. In many medical specialties, such as radiology, neurology, and dermatology, diagnosis is supported and based on images [12]. Automatic image diagnosis is probably the domain with more studies of medical artificial intelligence applications.

## 3. Ischemic Stroke and Artificial Intelligence: Are You a Bot? Please Select All Images Containing Ischemic Stroke

**Figure 1:**
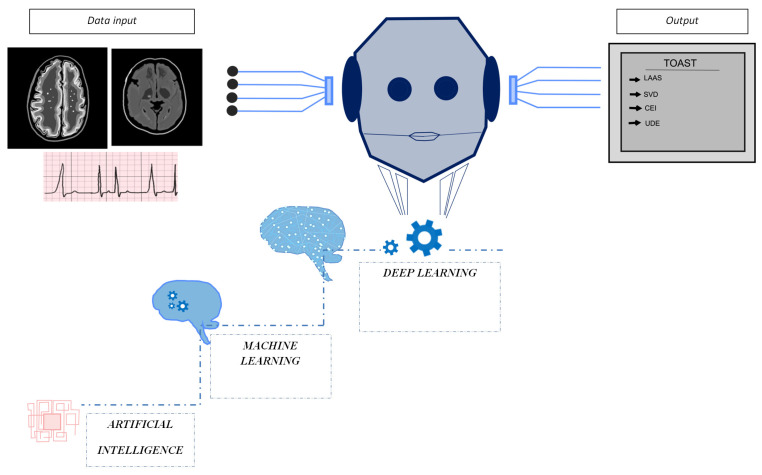
Artificial intelligence in stroke diagnosis according to TOAST classification. Artificial intelligence path over recent years has been a stairway to matching human complexity, introducing increasingly complex networks of data processing, among which deep learning is nowadays one of the leading figures. These complex algorithms allow for elaborate input data (e.g., MRI, ECG leads), which are acquired, read, and finally processed and interpreted. The possibility to elaborate data from ECG, MRI, CT images, and ultrasound allow for an elaborate algorithm for stroke subtype diagnosis.

Programs based on AI, such as ML, DL, and artificial neural networks (ANNs), have been applied in numerous studies evaluating the impact of AI in IS in different stages of care management, from prevention and diagnosis to rehabilitation and prognosis (Figure 1).

Prediction of IS in patients with atrial fibrillation (AF) represents one of the most studied issues. The use of ML and DL for electrocardiogram evaluation in the attempt to recognize occult flutter and AF is just an example of AI application with solid results in terms of accuracy and sensitivity of diagnosis [13]. Interestingly, several large-scale Asian studies [14,15,16] have used the ML approach to estimate stroke risk prediction in patients with AF. AI has also recently been employed to improve adherence to the guidelines on the use of anticoagulants in primary prevention. ML-powered clinical decision support, producing a warning for medical doctors responsible for patients with atrial fibrillation at thromboembolism risk, seemed to improve adherence to guidelines [17]. Regarding diagnosis, the discrimination of minor stroke and transient ischemic attack (TIA) from their mimics represents an important field of application of AI. One real-life helping study has effectively used ML to differentiate TIA and minor stroke from their mimics [18]. Moreover, ML has been used to recognize and differentiate ischemic stroke from clinical data [19] and to predict the modified Rankin Scale of 90 days to identify patients who need thrombectomy [20]. Undoubtedly, AI has found its maximum expression of potential with its application in diagnostic imaging exams. The employment of AI in large datasets obtained from imaging exams appears to be promising in overcoming the limit of heterogeneity of traditional qualitative interpretation performed by clinicians. In this context, ML is emerging as a notable approach to easily perform automatic diagnosis and image segmentation. In regard to LAAS prevention, Deep CNN has been successfully employed in carotid plaque ultrasound evaluation in order to predict plaque tissue rupture risk [21]. In addition, the application of CNN for an inexpensive exam like an intracranial ultrasound for the recognition of intracerebral stenosis demonstrated good sensitivity and specificity, overcoming the operator-dependent problems closely related to ultrasound examination [22]. In addition, several studies have employed ML in CTA imaging interpretation to detect stroke and large vessel occlusion [23,24]. Moreover, ML has also been shown to be promising in predicting and quantifying the ischemic core, starting with Computed tomography perfusion in the acute phase [23,25]. Based on MRI data, DL has shown good performance in classifying the time of onset of ischemic stroke [26] and phenotyping acute ischemic lesions according to the volume of lesions thanks to DL [27,28]. Furthermore, thanks to sophisticated algorithms developed on MRI images, some researchers focused on the study of white matter hyperintensities in order to recognize IS secondary to small intracranial vessels diseases and distinguish from other potentially similar conditions such as neurodegenerative diseases and multiple sclerosis [29,30]. Additionally, recognizing the correct etiology of IS according to TOAST classification leads to better management, therapeutic choice, and prognosis stratification of patients. In this regard, the development of an artificial neural network algorithm for the determination of TOAST subtypes by estimating probabilities for the main IS subtypes: atherothrombotic, cardioembolic, and lacunar, with high diagnostic and predictive significant results [31]. The complexity of the algorithm should require data from the results of computer tomography, duplex scanning of brain vessels, echocardiography, Holter monitoring, electrocardiography, and clinical estimation. The impact of AI has not been limited to the diagnostic process of ischemic stroke but, actually, prognosis studies represent one of the most frequent applications of ML especially focusing on the prediction of stroke-related risk of death [32,33]. Several studies have employed ML algorithms to predict functional outcomes of three months and recurrence after an ischemic stroke [34,35,36,37]. Finally, AI has been additionally applied to improve rehabilitation in an attempt to personalize rehabilitation programs and optimize resources and recovery time [38].

## 4. Research Strategy

A simple PubMed search of the keywords “Artificial Intelligence and stroke” yielded 699 publications. The first articles were published in the nineties but only twenty-six (26) publications before 2010 and 604 in the last five years. This trend demonstrated the growing interest and popularity of artificial intelligence applied to ischemic stroke. Since the rapid and continuous evolution in the last five years of this field with the introduction of new models and very different algorithms, the overall presentation was provided in terms of “narrative review”. In this review, our aim was to clarify the apport of artificial intelligence in ischemic stroke subtyping in accordance with TOAST classification. We conducted our research strategy on PubMed and Google Scholar to identify the most valuable evidence in the area of artificial intelligence applied to the etiologic diagnosis of stroke. We take into major consideration articles published in the last 5 years. The initial research was improved by introducing some keywords: “TOAST classification”, “stroke subtyping”, “carotid stenosis”, “atrial fibrillation”, “cardioembolic stroke”, “large artery stroke”, “small vessel disease”, “ESUS”, “PFO”, “stroke of unknown origin”. The references from the research were also shortlisted to complete the structure of this review. This narrative review is the results of more than 100 studies considering the application of AI to diagnostic subtyping of ischemic stroke that were meticulously screened by experts in the field. Since the objective of this review is to provide the broadest and most comprehensive knowledge of the tools that exploit artificial intelligence for the etiological diagnosis of ischemic stroke, the selected publications concerning the applications of models and algorithms to first-level diagnostic tests have been considered, such as ultrasound and electrocardiogram as well as second level diagnostic tests such as computed tomography and magnetic resonance.

## 5. Results

The possible causes of ischemic stroke can be multiple, and often, two or more potentially responsible conditions can be present simultaneously in the same patient. Therefore, it is not always easy to identify the correct etiological diagnosis starting from the characteristics of the ischemic lesion found on brain CT and the patient’s comorbidities. Generally, in clinical practice, this diagnostic process involves the execution of several instrumental tests aimed at excluding or confirming the presence of relevant conditions for the identification of the cause. Despite this, the percentage of strokes classified as UDE is around 20–40% of all cases. The use of artificial intelligence with the intrinsic ability to process numerous data to identify connections that cannot be detected by clinical investigations is finding more and more space in this field. At the moment, however, not many quality studies have applied large-scale AI models considering data from multiple diagnostic tests, even heterogeneous, such as ECG-Holter for the identification of atrial fibrillation, ultrasound of the carotid artery for the diagnosis of stenosis and occlusion of the cerebro-afferent vessels and MRI for identification of small vessel disease. In fact, most studies have focused on the characterization of a single TOAST subtype. For this reason, the results of this review are presented below using a subdivision that follows the main TOAST subtypes: LAAS, Cardioembolic, SVD, and UDE.

### 5.1. Large-Artery Atherosclerosis Subtype

Detection of large vessel occlusion is one of the most pursuable objectives in applying AI to cerebrovascular pathology. Furthermore, early recognition of LVO using AI can be helpful in triage, diagnosis, patient selection for treatment, and stratification of the prognosis in acute cerebrovascular disease. In recent years, the increasing use of artificial intelligence through Computer-aided diagnosis (CAD) systems provides computational methodologies for quantitative and objective evaluations, eliminating as far as possible individual mistakes and increasing the diagnostic sensitivity in atherosclerotic disease [39,40]. The application of AI in diagnosing LVO aims to identify atherosclerotic lesions in the carotid arteries and the middle cerebral artery. Using CAD based on CNNs (convolutional neural networks) allows a more accurate assessment of carotid disease. The CNNs approach provides a classification model by assessing medical images to derive objective parameters through convolutional and fully-connected layers able to detect particular patterns and obtain an output layer in an automated way for future prediction. In these models, inputs from convolutional layers converge through a rectified linear unit, later selected from a max pooled layer responsible for downsampling to decrease the spatial dimension of each feature map and finally directed to fully connected layers. A final dropout layer allows for avoiding overfitting. The softmax function calculates the final output as a stroke if the probability is more than 50%, providing a binary method for classifying the events. Several CNN models have been created for this purpose. One of the first devices designed was AlexNet, constituted of five convolution layers, three max-pooling layers, and three fully connected layers [41].

More accurate equipment has been recently developed. Inception-v3 reduces the computational complexity using a multipath structure involving dimensional reduction and parallel structures [42]; ResNet adds a skip connection where the gradients can cross the network [43], while DenseNet links the output of each layer to the input of the following layer through transition layers, which deliver fewer feature maps than those received [44]. In one study by Agedpchung-Ming Lo et al., the use of CNN models using convolutional, pooling, and fully connected layers has been proposed to identify diagnostic features from the carotid color Doppler (CCD) study. Final results showed a significant accuracy, sensitivity, and specificity of all the CNN architectures, suggesting the possible role of the CAD system for predicting the risk of ischemic cerebrovascular events using an automatic and standardized method, even if more studies must be carried out [45]. These data were confirmed by a more recent study [46] evaluating the feasibility of the application of a CNN architecture for diagnosing critical carotid stenosis building on the NASCET US criteria. The algorithm showed a high sensitivity, specificity, and accuracy in detecting normal carotid artery (91%, 86%, and 92%) and critical carotid stenosis (92%, 87%, and 94%), demonstrating the applicability of the AI in the diagnosing carotid artery disease in greyscale static DUS images and providing the possibility to non-expert to diagnose carotid disease.

AI has been recently applied to create a machine-learning tool using CTA images for evaluating intracranial internal carotid artery (ICA) stenosis in patients with acute ischemic stroke. StrokeSENS LVO model has been created by analyzing retrospectively 400 studies (217 LVO, 183 other/no occlusion): the algorithm has shown a high accuracy (92.7%), sensitivity (85.7%), and specificity (87.4%) in detecting intracranial ICA occlusion, without differences in patient age, sex, or CTA acquisition characteristics [47]. Moreover, Buckler proposed an interesting deep-learning algorithm for the stratification of atherosclerotic lesions in different phenotypes based on plaque stability [48]. The results were supported by histopathological confirmation, proposing this model for detecting the carotid lesions more susceptible to embolization or thrombosis and thus at high risk of ischemic stroke. Hyperattenuating artery signal in M1 MCA is a representative sign of a thrombotic event, detectable in the early phases of cerebral ischemia [49,50,51]. If the hyperattenuating sign is detectable in the segmental branch of MCA (M2-M3) within the Sylvian fissure, it is called the “MCA dot sign” and is associated with a better prognosis [52,53].

Takahashi et al. [54] proposed an SVM (support vector machine) model to detect MCA dot signs based on the extraction of NCCT—images from the sylvan fissure region, where the middle cerebral artery is located. The automated system eliminates false positive signals, identifying the group of patients with MCA dots. In the study, 39 of the 40 patients with acute stroke and occlusion of the MCA were identified, while one was indicated as a false positive. In addition, the system could identify people without MCA dots, eliminating 271 false positive patients from the analysis. These data underlined the high sensitivity of the method (97.5%) in identifying MCA dots, even if the study was affected by some limitations, such as the small size of the database, the evaluation on one hemisphere, and the definition of the used parameters in an empirical manner.

Furthermore, You et al. proposed a hierarchical modeling based on three levels for predicting LVO stroke resulting from MCA occlusion. Level-1 relies on the demographic features and clinical signs, while Level-2 is based on evaluating pre-existing medical conditions predisposing to cerebral ischemia. Level-3 consists of fully conventional networks (XGBoost) belonging to a deep learning model comprehensive of an encoding part, which extracts images from NCCT, and a decoding part able to reconstruct the segmentation label map. Compared to SVM, Random Forest, and logistic regression, XGBoost showed higher accuracy (80.0%), sensitivity (95.3%), and specificity (68.4%) in detecting MCA occlusion directly, independently from the presence of the hyperdense MCA sign, which is an LVO marker but whose absence does not allow to exclude MCA occlusion [55].

Moreover, a fascinating machine-learning model has been proposed to recognize the source of a clot in MCA based on its characteristics [56]. The algorithm was created considering pre-endovascular treatment gradient echo (GRE) MRI images in patients with middle cerebral artery occlusion. It was able to select patients with occlusion related to atrial fibrillation with high accuracy. Furthermore, patients with atrial fibrillation had a better response to endovascular treatment using a stentriever and less probability of restenosis than patients with intracranial atherosclerosis; in this concern, this model may be helpful to guide the most appropriate treatment to ensure faster and prolonged recanalization. The importance of early detection of MCA dot signs is that this signal appears on CT images before the finding of hypoattenuation of the ischemic stroke regions. For this reason, AI can be helpful to indirectly diagnose ischemic events which segmentation CAD models could not detect, accelerating access to treatment. Even if initial evaluation in The Automated Large Arterial Occlusion Detection IN Stroke Imaging (ALADIN) trial led to few results in the use of AI for the identification of LVO starting from CT images [57], a computer system approved by FDA in 2018, called Viz. AI Contact and used by Barreira et al. in a patient cohort from the ALADIN trial, proved to be a good predictor for artery occlusion with high sensitivity, specificity, and accuracy (90.1%, 82.5%, and 86%, respectively) [57]. The use of Viz. AI model has been associated with the reduction of 22.5 min of the transfer times to adequate stroke units, positively affecting complications and the overall outcome after vessel occlusion [58]. Compared with Viz.AI, RAPID.AI, and Brainomix algorithms showed similar accuracy in detecting all LVOs (71% and 77%, respectively), especially in the case of M1 occlusion where the sensitivity of the models reached higher percentages (83% and 94%, respectively) [59].

In a recent study performed in 2021 by Ryan et al., ^AUT^Canon’s Stroke Solution LVO application has shown a high specificity and sensitivity in detecting ICA occlusion, while it has demonstrated lower sensitivity in the diagnosis of M1 MCA occlusion. Moreover, Canon’s software showed a high negative predictive value (84%) in case of clot lack in M1 MCA, allowing to rule out patients not requiring thrombectomy. Furthermore, the sensitivity of the software decreased with the vessel size reduction, demonstrating the poor ability to detect M2 MCA occlusion [60]. The application of AI in predicting the outcome of patients with acute ischemic stroke concerns not only the early detection of clots into large vessels, which represents the stroke subtype more related to disability, but also the identification of patients at increased risk of developing complications in this population. Ding et al. have proposed the creation of a deep neural network -model based on six variables from the Acute Stroke Registry to predict a 3-month mRS score better than the traditional clinical scores (Acute Stroke Registry, Analysis of Lausanne score) [61]. Other studies confirm the benefit of applying ML algorithms built on factors such as small infarct core, NIHSS score after 24 h, premorbid mRS score, and infarction volume on post-interventional CT to prognosticate the functional outcome after acute vessel occlusion [62,63,64,65,66]. Evaluating the extension of the ischemic core and vital tissue in the brain lesions helps define the right strategy for treatment. The ASPECT score is traditionally used to estimate lesion segmentation in the case of acute middle cerebral artery stroke involving two strategic cerebral regions, the basal ganglia plane, and the supraganglionic plane. It is a 10-point numerical topographic CT score obtained, detracting one point from the total for any sign of ischemic signals [67].

Starting from this scoring system, some automated software (RAPID ASPECT score, Frontier ASPECT Score Prototype, and e-ASPECTS Brainomix software) have been generated and have been recognized to have similar accuracy in detecting the difference between ischemic and non-ischemic brain tissue in comparison with expert neuroradiologists [68,69,70]. The e-ASPECTS Brainomix software uses a deep learning classification to generate an ASPECT score with a sensitivity of 83%, even if the expert evaluation should be recommended in case of pre-existing cerebral abnormalities [71]. Since vessel occlusion leads to the diversion of the blood flow, activating circles of compensation, collateral circulation is one evaluation to define the reperfusion treatment candidacy. The combination of classical images from CTA and ML classifiers has been used to create modules for the rapid and automatic identification of collateral flow, with similar accuracy to a consensus expert CTA–collateral scoring approach [72,73]. Finally, intracranial hemorrhage is a potential complication of thrombolytic treatment, so evaluating the risk may guide the choice of recourse to reperfusion therapy. In this concern, starting from data obtained from MR perfusion and DWI, machine learning algorithms have been created to predict the risk of bleeding after thrombolysis with an accuracy of 83.7% [74].

### 5.2. Cardioembolic Source Detection

The cardioembolic ischemic stroke subtype embraces patients with arterial occlusions due to embolus emerging from the heart. Thus, at least one cardiac source for an embolus must be identified to confirm the diagnosis [2]. Cardioembolic stroke accounts for 14–30% of all cerebral infarctions [75,76], and despite advanced therapies for dyslipidemia and arterial hypertension, it constitutes a rising source of stroke in wealthy nations. In particular, AF is a common arrhythmia and determines the increased risk of stroke and systemic embolism [77]. Although AF diagnosis requires simple tools (electrocardiography (ECG) documentation), the screening is often challenging, especially in patients with paroxysmal AF due to the frequent absence of symptoms [78]. Although since the 1900s, initially promising computer programs have accomplished an automated analysis of ECGs’ patterns, their performance is now notably inconsistent and full of inaccuracies [78]. AI and, in particular, ML provide systems with the ability to learn from data and thus escape the limitations of common automated computerized ECG interpretation programs [78]. Recently, Attia et al. [79] demonstrated that an AI-ECG algorithm, developed by using more than 500,000 normal sinus rhythm standard 12-lead ECGs from over 180,000 patients, had a strong potential for detecting patients with a high likelihood of paroxysmal AF or atrial flutter suggesting a potential and substantial change in stroke risk assessment and management. DL is perhaps the most commonly used technique for ECG analysis and interpretation [78]. In particular, CNNs are more and more widely used in automated ECG classification [80]. As already described, CNN is a model that is generally used to analyze data that has a grid pattern, such as an image, and is designed to learn spatial hierarchies of features. At the end of the process, it is possible to classify objects in an image [81], such as ECG patterns. Several researchers have studied neural networks’ ability to detect cardiac arrhythmias, mainly by using a single-lead source and rarely a 12-lead source [80,82,83,84,85]. However, AI-ECG has its own limitations: (i) quality of data comes from the signal acquisition, which is operator-dependent, intrinsically associated with human error, and environment-dependent, which can be noisy [86]. (ii) AI-ECG algorithms are trained on homogenous populations; hence the risk of generalization since the impact of race and ethnicity on ECG analysis via ML is actually unknown. Large volume data from diverse demographics will be helpful in order to define a more accurate and tailored application of models. Furthermore, ML was successfully used to perform cardioembolic stroke subtyping using an electronic health record database [87]. The best model presented an accuracy of 92.2%, identifying atrial fibrillation, age, dilated cardiomyopathy, congestive heart failure, patent foramen ovale, mitral annulus calcification, and recent myocardial infarction as the main discriminatory features. Moreover, the majority of the abovementioned studies have taken into account only one of twelve leads, while it would be desirable in the future to develop CNNs able to accurately process more data.

Interestingly, some authors tried to classify cardioembolic stroke by applying DL to a simple diagnostic test like chest radiography [88]. The final model demonstrated good classification feasibility and biological plausibility in differentiating cardioembolic versus non-cardioembolic stroke with a better performance in high-risk sources such as AF.

Moreover, a recent study used ML to analyze echocardiographic and cardiac resonance imaging parameters in left ventricular non-compaction cardiomyopathy to find the best predictors of clinically relevant outcomes, including cardio embolism and stroke [89]. Finally, AI has also been used to clarify the origin of IS with cardioembolic sources in particular populations like in Chagas Disease [90]. Authors built a sensitive predictive model for cardioembolic stroke classification in Chagas disease using Random Forest methodology. This hierarchical model of decision tree appears to be worthy of interest thanks to its predictive capacity and pragmatic problem-solver methodology in the case of classification.

### 5.3. Small Vessel Disease Identification

Cerebral small vessel disease (cSVD) is a definition that gathers different pathological processes that affect the small vessels of the brain (small arteries and veins and capillary beds) whose occlusion is responsible for a specific ischemic stroke subtype referred to as “lacunar stroke”. It accounts for a quarter of ischemic strokes and represents a major cause of vascular dementia [91,92]. In current clinical practice, MRI is used to make a diagnosis by evidencing common findings in cSVD patients, such as white matter hyperintensities, lacunar infarcts, microbleeds, perivascular spaces (PVS), and cerebral atrophy, whose evidence is considered diagnostic for cSVD [93]. However, since medical image interpretations require human observers, they are linked to biases and variations which come from human error and subjectivity. Recently, CNN was found to be useful both in the identification of cSVD markers and stratification of cSVD (low, medium, and high severity), representing an instrument able to overcome flaws intrinsic in human-related neuronal processes. Lambert et al. [94] studied an automated white matter lesion segmentation algorithm to assess the severity of cSVD. Ciulli et al. [95] proposed a machine-learning algorithm tailored to investigate the link between alterations in executive functions in patients with mild cognitive impairment and cSVD and to associate them with the brain substrates of this impairment. Furthermore, interest has recently grown in PVS evaluation. PVS, also known as Virchow–Robin spaces, are spaces filled with interstitial fluid that encompass small brain vessels and capillaries following their path inside grey or white matter and act as a metabolic drainage system for the brain. When they are enlarged, they are visible in MRI sequences [96], appearing as microscopic (less than 3 mm diameter) linear or dot-like structures with intensity similar to the cerebrospinal fluid [93]. Although potentially quantifiable, PVS visual counting and delineation can be time-consuming even applying computational qualitative and quantitative methods available, which are still semi-automatic and hence observer-dependent [30,97].

In this regard, González-Castro et al. [98] proposed an automatic scheme to qualitatively classify T2-weighted MRI (as having none or few PVS compared with having many of them) in the basal ganglia region and comparing their results with visual ratings made by an experienced neuroradiologist and by a trained image analyst showing that the goodness-of-fit of the model for the automatic classifier was good. Additionally, in a retrospective analysis conducted on 1156 patients, a deep-learning system for automatic prediction of white matter hyperintensities on FLAIR images demonstrated good accuracy with a smaller analysis time for physicians [98].

During the past few years, a possible role of neutrophils containing cytotoxic aggregates has been suggested to be a causative element in endothelial dysfunction, BBB disruption, and ischemic brain injury [99,100]. Therefore, markers of neutrophil activation, such as myeloperoxidase and calprotectin, could help both to ascertain neutrophils’ role in cSVD pathogenesis and to identify cSVD patients. Several studies have shown a relationship between endothelial dysfunction and WMH and lacunar infarction [101,102,103]. Recently, Karel et al. [92] investigated levels of markers reflecting neutrophil activation, neutrophil extracellular trap (NET) formation, platelet activation, and vascular inflammation in plasma samples of cSVD patients and controls. In order to identify differences in patients’ and controls’ myeloperoxidase and other marker levels, machine-learning technology was applied and implemented. They found that among above mentioned markers, only myeloperoxidase levels were altered, and this element was considered an important feature in the detection and prediction of cSVD. Wang et al. [104] assessed the prognostic ability of SVD imaging markers on acute ischemic stroke subtypes using machine learning and logistical regression methods and found that in lacunar stroke patients, models using SVD imaging markers could rapidly predict prognosis. Nevertheless, the mechanism by which SVD affects the prognosis of acute ischemic stroke patients is poorly understood. 

### 5.4. Stroke of Undetermined Etiology

Underlying pathologies of stroke of unknown cause include occult paroxysmal AF, patent foramen ovale (PFO), aortic arch atheroma, and thrombophilia. AF misdiagnosis burdens stroke diagnostic management since it accounts for 8–15% of embolic stroke of an undetermined source (ESUS) cases [105,106]. ESUS has a strong social and healthcare burden since 1 in 20 patients each year experience a recurrent stroke, which is often more severe than the first one [107,108]. Despite the number of diagnostic tools available, ESUS often remains unlabeled with an inevitable impact on secondary prevention and focused treatments to prevent recurrent stroke after ESUS. The detection rate of AF has raised since methods of prolonged ambulatory cardiac rhythm monitoring devices have been introduced to the diagnostic tools at our disposal. Still, paroxysmal AF is identified only in a few numbers of patients with ESUS, even after continuous monitoring for three years. This missed diagnosis cannot be outclassed by directly pursuing anticoagulation therapy regardless of AF detection. In fact, RCTs comparing direct oral anticoagulants with antiplatelet therapy in patients with ESUS did not show benefit from anticoagulation, implying that a definite diagnosis must be made before embracing anticoagulant therapy [106,107,109]. Thus, identifying undiagnosed paroxysmal AF in ESUS has a therapeutic advantage, and the development of tools able to accomplish this difficult task would represent an important step for these patients’ outcomes. Recently, Kamel H. et al. [110] analyzed 1663 ischemic stroke patients registered in Cornell AcutE Stroke Academic Registry (CAESAR) from 2011 to 2016 and trained an ML algorithm to distinguish cardioembolic from non-cardioembolic ESUS associating the predicted probability of a cardioembolic source with the eventual post-ESUS diagnosis of AF. Among 580 ESUS patients, the estimated likelihood of an occult cardioembolic source, calculated by a machine-learning algorithm, was associated with the detection of AF [110]. Interestingly, a study conducted on 800 consecutive ESUS patients applied a hierarchical k-means clustering algorithm for categorizing potential embolic sources [111]. This ML analysis identified four different clusters of patients associated respectively with arterial disease, atrial cardiopathy, PFO, and left ventricular disease, with a clear prevalence of the first group.

Continuing on ESUS, a recent multi-center study by Luo et al. [112] analyzed a small sample of patients affected by an embolic stroke of unknown source and PFO using unsupervised hierarchical clustering to detect sub-clusters in post-closure PFO patients and identifying predictors for adverse outcome. They found that traditional cardiovascular risk factors remain the best predictors for recurrent stroke and TIA in post-closure PFO patients. 

Finally, in a fascinating recent study [113], authors performed an ML cluster analysis of frequent biomarkers in subjects admitted with severe acute respiratory syndrome coronavirus 2 to determine if any were associated with acute ischemic stroke. They conclude that excess prevalence of acute IS in patients with COVID-19 could be associated with COVID-19 severe respiratory disease or coagulopathy, suggesting that COVID-19-associated ischemic stroke may be defined as the otherwise cryptogenic cause after full diagnostic evaluation in the setting of severe systemic COVID-19 disease or COVID-19–associated coagulopathy as identified by an elevated D-dimer burden.

## 6. Discussion

### 6.1. AI and LAAS

The use of AI in the detection of LVO is an essential tool in the management of acute cerebrovascular disease. Early recognition of LVO using AI can aid in triage, diagnosis, patient selection for treatment, and stratification of prognosis. CAD systems provide computational methodologies for objective evaluations, increasing diagnostic sensitivity in atherosclerotic disease. The CAD models based on CNNs allow for accurate assessments of carotid disease. Several CNN models, such as AlexNet, Inception-v3, ResNet, and DenseNet, [41,42,43,44] (Table S1), have been developed to classify medical images and derive objective parameters for future predictions. These models have shown comparable high sensitivity, specificity, and accuracy in detecting normal and critical carotid stenosis. AI has also been applied to create a machine-learning tool using CTA images for evaluating intracranial internal carotid artery stenosis in patients with acute ischemic stroke. The machine-learning tool called the StrokeSENS LVO model was created using CTA images to evaluate intracranial internal carotid artery (ICA) stenosis in patients with acute ischemic stroke [47]. The algorithm showed high accuracy, sensitivity, and specificity in detecting intracranial ICA occlusion, without differences in patient age, sex, or CTA acquisition characteristics. Furthermore, a deep-learning algorithm has been proposed for stratifying atherosclerotic lesions in different phenotypes based on plaque stability, which could identify carotid lesions susceptible to embolization or thrombosis and thus at high risk of ischemic stroke [48]. One study by Agedpchung-Ming Lo et al. [45] used CNN models to identify diagnostic features from carotid color Doppler images. The results showed a significant accuracy, sensitivity, and specificity of all the CNN architectures, suggesting the possible role of the CAD system in predicting the risk of ischemic cerebrovascular events. These interesting results have been confirmed by another study that evaluated the feasibility of applying a CNN architecture for diagnosing critical carotid stenosis using greyscale static DUS images [46]. The algorithm showed a high sensitivity, specificity, and accuracy in detecting normal and critical carotid stenosis, demonstrating the applicability of AI in diagnosing carotid artery disease. Moreover, regarding the hyperattenuating artery signal in M1, Takahashi et al. [54] proposed an SVM model to detect MCA dot signs based on the extraction of NCCT images from the sylvan fissure region where the middle cerebral artery is located. The automated system eliminates false positive signals, identifying the group of patients with MCA dots. The system showed a high sensitivity (97.5%) in identifying MCA dots. However, some limitations, such as the small size of the database, the evaluation of one hemisphere, and the empirical definition of parameters, affect the study. Interestingly, hierarchical modeling based on three levels has been proposed for predicting LVO stroke resulting from MCA occlusion [55]. Level-1 relies on demographic features and clinical signs, Level-2 evaluates pre-existing medical conditions predisposing to cerebral ischemia, and Level-3 considers the imaging features of NCCT and CTA. This model has demonstrated high accuracy in predicting LVO stroke. 

Overall, the abovementioned models have shown promising results in aiding the diagnosis of cerebrovascular disease, specifically LVO. However, more studies are needed to validate the accuracy of the models in a larger and more diverse patient population. 

### 6.2. AI in Cardioembolic Stroke

Cardioembolic stroke is a significant cause of cerebral infarctions and is a rising source of stroke in wealthy nations. AF is a common arrhythmia and increases the risk of stroke and systemic embolism. The evidence presented in this review suggests that AI and ML have the potential to revolutionize stroke risk assessment and management, especially in detecting patients with a high likelihood of paroxysmal AF or atrial flutter (Table S2). Attia et al. demonstrated the strong potential of an AI-ECG algorithm, developed using more than 500,000 normal sinus rhythm standard 12-lead ECGs from over 180,000 patients, to detect patients with a high likelihood of paroxysmal AF or atrial flutter [79]. Our results highlight the limitations of AI-ECG, including the quality of data, which comes from signal acquisition and is operator-dependent, intrinsically associated with human error, and environment-dependent, which can be noisy. Additionally, AI-ECG models are developed on homogenous populations, which increases the risk of generalization since the impact of race and ethnicity on ECG analysis via ML is unknown [86]. According to the results of this review, DL is perhaps the most commonly used technique for ECG analysis and interpretation. CNNs are more and more widely used in automated ECG classification and can accurately classify ECG patterns. ML was successfully used to perform cardioembolic stroke subtyping using an electronic health record database [87]. The best model identified atrial fibrillation, age, dilated cardiomyopathy, congestive heart failure, patent foramen ovale, mitral annulus calcification, and recent myocardial infarction as the main discriminatory features. Interestingly, some authors tried to classify cardioembolic stroke by applying DL to a simple diagnostic test, like chest radiography. The final model demonstrated good classification feasibility and biological plausibility in differentiating cardioembolic versus non-cardioembolic stroke with a better performance in high-risk sources such as AF. In conclusion, AI and ML have the potential to aid in the diagnosis and management of cardioembolic ischemic stroke. While there are limitations to these technologies, research has shown promising results in detecting cardiac arrhythmias, classifying cardioembolic stroke, and predicting clinically relevant outcomes. Further research is needed to overcome the limitations of these technologies and improve their accuracy and generalization.

### 6.3. AI in Small Vessel Disease

In this review, we have discussed the use of different models to diagnose and predict cSVD. While MRI is commonly used in clinical practice to diagnose cSVD, it is limited by the subjectivity and bias of human observers. To overcome these limitations, researchers have turned to machine learning and deep learning models. Several studies have shown the effectiveness of these models in identifying cSVD markers, stratifying cSVD severity, and predicting prognosis in patients with lacunar stroke (Table S3). Lambert et al. [94] used an automated white matter lesion segmentation algorithm to assess the severity of cSVD, while Ciulli et al. [95] proposed a machine-learning algorithm to investigate the link between cSVD and executive function impairments. PVS has also been the focus of research. Although potentially quantifiable, manual counting and delineation of PVS can be time-consuming and observer-dependent. González-Castro et al. [98] proposed an automatic scheme to qualitatively classify T2-weighted MRI based on PVS in the basal ganglia region, demonstrating good results. Finally, markers of neutrophil activation, such as myeloperoxidase and calprotectin, have also been investigated to identify cSVD patients. Karel et al. [92] used machine learning technology to identify differences in myeloperoxidase levels between cSVD patients and controls, suggesting its potential as an important feature in detecting and predicting cSVD. Overall, the use of machine learning and deep learning models in cSVD diagnosis and prognosis prediction holds promise in overcoming the limitations of human observation and improving accuracy and efficiency.

### 6.4. Stroke of Unkown Origin and AI

One of the most interesting data discussed in this review derived from the analysis of different models for strokes of unknown cause, including paroxysmal AF, PFO, aortic arch atheroma, and thrombophilia. The misdiagnosis of AF as the cause of stroke burdens diagnostic management and may lead to ineffective treatments. ESUS, which accounts for a significant portion of embolic stroke cases, has a strong social and healthcare burden due to the high risk of recurrent stroke, which is often more severe than the first. Despite the number of diagnostic tools available, ESUS often remains unlabeled, making secondary prevention and focused treatments challenging. Recent studies have applied machine learning algorithms to identify potential embolic sources and predict the likelihood of an occult cardioembolic source associated with AF (Table S4). For instance, Kamel et al. [110] trained an ML algorithm to distinguish cardioembolic from non-cardioembolic ESUS and associate the predicted probability of a cardioembolic source with the eventual post-ESUS diagnosis of AF. Hierarchical k-means clustering algorithms have also been used to categorize potential embolic sources, identifying four different clusters of patients associated with arterial disease, atrial cardiopathy, PFO, and left ventricular disease [111]. Furthermore, a recent multi-center study analyzed a small sample of patients affected by an embolic stroke of unknown source and PFO using unsupervised hierarchical clustering to detect sub-clusters in post-closure PFO patients and identify predictors for adverse outcomes [112]. The study found that traditional cardiovascular risk factors remain the best predictors for recurrent stroke and TIA in post-closure PFO patients. Since the great interest in COVID-19 coagulopathy, we reported some interesting data about the excess prevalence of acute IS in patients with COVID-19 [113]. This could be associated with COVID-19 severe respiratory disease or coagulopathy, suggesting that COVID-19-associated ischemic stroke may be defined as otherwise cryptogenic cause after full diagnostic evaluation in the setting of severe systemic COVID-19 disease or COVID-19-associated coagulopathy as identified by an elevated D-dimer burden. Overall, the abovementioned models aim to improve the diagnosis and management of stroke of unknown cause, which has a strong social and healthcare burden. The development of tools able to identify undiagnosed paroxysmal AF in ESUS patients would represent an important step in improving patient outcomes.

## 7. Conclusions

Artificial intelligence through ML has proven to be a useful tool for identifying predictive factors capable of subtyping acute stroke patients in large heterogeneous populations. Additionally, AI seems able to clarify the etiology of recurrent strokes of unknown origin, especially in detecting cardioembolic sources. Thus, more studies are necessary to validate AI techniques and models before they can spread and be used in clinical practice. However, the extensive use of diagnostic images analyzed through DL models seems to favor the diagnostic and prognostic role of instrumental tests to the detriment of the clinical information. Thus, some important issues need to be addressed. First, it is desirable that clinicians who will have to take clinical decisions on the basis of AI model results are able to interpret the algorithms by understanding their limits to not incur serious errors of inaccuracy. Furthermore, future studies should integrate the algorithms oriented toward diagnostic imaging with essential clinical information to increase their reliability and accuracy in clinical practice.

## Data Availability

The data and images used in the current study are available from the corresponding author upon reasonable request.

## Supplementary Materials

The following supporting information can be downloaded at: https://www.mdpi.com/article/10.3390/biomedicines11041138/s1, Table S1: Application of AI in the diagnosis of LAAS stroke. Table S2: Application of AI in cardioembolic source detection. Table S3: Main studies considering application of AI in Small Vessel Disease diagnosis. Table S4: AI application in stroke of undetermined etiology.

## Abbreviations

AF: atrial fibrillation; AI: Artificial intelligence; ANNS: artificial neural networks; CAD: Computer-aided diagnosis; CCD: carotid color Doppler; CCNs: Convolutional neural networks; CE: Cardioembolism; CTA: computed tomographic angiography; DL: Deep learning; ESUS: embolic stroke of an undetermined source; IS: Ischemic stroke; ICA: Internal carotid artery; LAAS: large-artery atherosclerosis; LVO: Large vessel occlusion; MCA: middle cerebral artery; ML: machine learning; MRI: magnetic resonance imaging; NCCT: Non-contrasted computed tomography; NET: neutrophil extracellular trap; NN: Neural networks; ODE: stroke of other determined etiology; PFO: patent foramen ovale; PVS: perivascular spaces; cSVD: Cerebral small vessel disease; SVM: support vector machine; TIA: Transient ischemic attack; UDE: stroke of undetermined etiology; US: ultrasound.
