# Supplementary material for: Artificial Intelligence in Acute Ischemic Stroke Subtypes According to Toast Classification: A Comprehensive Narrative Review
Source: Biomedicines. 2023 Apr 10;11(4):1138. doi: 10.3390/biomedicines11041138 (PMC10135701; doi:10.3390/biomedicines11041138)
Supplement: Supplementary file 1 [file biomedicines-11-01138-s001.zip › biomedicines-2280597-supplementary.pdf]

Supplementary Materials

Table S1. Application of AI in the diagnosis of LAAS stroke.

References	AI-based approaches	Data collection	Study aims and clinical implications	Results
Agedpchung-Ming et. al (2021)	CNNs (*AlexNet*)	1032 CCD images (106 patients with acute ischemic stroke:549 CCD images, and 79 normal controls: 483 CCD images)	Proposing a CCD-images based on automated system for evaluating carotid stenosis for prediction of the risk of acute ischemic stroke	**Accuracy** 91.67% **Sensitivity** 93..33% **Specificity** 90.20% **AUC** 0.9432
Kordzadek et al. (2022)	CNNs	156 images of normal and stenotic carotid arteries (based on NASCET criteria)	Recognition of carotid artery disease through an automated algorithm using greyscale static DUS images	**Accuracy** 92% **Sensitivity** 91% **Specificity** 86%
Cimflova et al. (2022)	ML (*StrokeSENS LVO Detection*)	400 CTA studies (217 LVO and 183 no occlusion)	Detection of anterior LVO using a machine-learning system based on the tomographic images of carotid arteries and M1 middle cerebral artery	**Sensitivity** 89.4% (overall), 85.7 (ICA), 91.4% (M1 MCA) **Specificity** 87.4% (overall), 87.4 (ICA), 87.4% (M1 MCA) **AUC** 0.939 (overall),0.927 (ICA), 0.945 (M1 MCA)
Buckler et al. (2023)	ML	904 CTA images (23 patients with atherosclerotic plaques:408 images, and 30 controls:496 images)	Recognition of carotid disease and lesion vulnerability evaluating the characteristics of the plaques on the CTA studies, confirmed through hystopatological analysis	**AUC** 0.97 (unstable plaque), 0.95 (stable plaque), 0.99 (minimal disease)
Takahashi et al. (2014)	SVM system	297 CT images (7 patients with MCA dot sign at the CTA study)	Detection of MCA occlusion recognizing dot sign on unenhanced CT images in patients with acute ischemic stroke	**Sensitivity** 97.5%
You et al. (2020)	ML (*XGBoost model*)	300 patients with acute ischemic stroke (130 patients with LVO)	Early diagnosis of LVO in acute setting using NCCT images-based on algorithm to decide best therapeutic approach	**Accuracy** 80% **Sensitivity** 95.3% **Specificity** 68.4%
Chung et al. (2019)	ML	67 patients with acute cerebral artery occlusion, whose 43.3% (29 patients) had AF (13 with known AF and 16 with new diagnosed AF)	Recognition of middle cerebral artery occlusion using MRI-based on automaded ML system to identify the cause of acute ischemic stroke (AF or atherosclerotic disease) analyzing clot composition for optimal therapeutic strategy (MT vs anticoagulation)	**Accuracy** 75.4% **Sensitivity** 79% **Specificity** 63% **AUC** 0.87
Barreira et al. (2018)	CNNs (*Viz-Al-Algorithm*)	Retrospective cohort of 875 AIS patients admitted to tertiary stroke centers from 2014 to 2017	Creation of an automated system using CTA images for detecting ICA or MCA-M1	**Accuracy** 86% **Sensitivity** 90.1% **Specificity** 82.5% **AUC** 0.863

			occlusion vs non-LVO ischemic stroke	
Hassan et al. (2020)	ML system (*Viz.AI*)	43 patients with clinical signs of acute ischemic stroke	Estimation of the reduction of the transfer time to a CSC to improve the outcome of patients early recognizing LVO using a CTA-imaging automatic technology	Reduction of time transfer to CSC with an avarage of 22.5 min (135 min versus 110 min; p=0.0470)
Mallon et al. (2022)	CNN (*Brainomix and RapidAI*)	90 patients with AIS, of which 64 had an LVO on CTA study (62 patients with ICA or MCA occlusion, 2 patients with basilar occlusion)	Correlation between the estimates of ischemic core and penumbra using CTP imaging softwares in association with clinical criteria (ASPECTS) for selecting patients with LVO elegible for mechanical thrombectomy	**Accuracy** 77% (Brainomix), 71% (RapiAI) **Specificity** 97% (Brainomix), 77% (RapidAI) **AUC** 0.65
Rava et al. (2021)	DL system (*Canon's AUTO Stroke Solution*)	303 AIS patients (202 with LVO and 101 with non-LVO stroke)	Rapid identification of ICA and MCA M1 occlusion by automation platform using CTA scans to select patients elegible for reperfusion strategies for improving clinical outcomes and reducing residual disabilities	**Accuracy** 81% (overall), 95% (ICA), 89% (MCA M1), 80% (MCA M2) **Sensitivity** 73% (overall), 90% (ICA), 77% (MCA M1), 51% (MCA M2) **Specificity** 98% (overall), 98% (ICA), 98% (MCA M1), 98% (MCA M2)

AF: atrial fibrillation; AI: artificial intellgence; AIS: acute ischemic stroke; ASPECTS: Alberta stroke program early CT score; AUC: area under the curves; CCD: carotid color Doppler; CNNs: convolutional neural networks; CSC: comprehensive stroke centers; CTA: computed tomographic angiography; CTP: computed tomography perfusion; DL: deep-learning; DUS: Doppler ultrasound; ICA: internal carotid artery; LAAS: large artery atherosclerosis; LVO: large vessel occlusion; MCA : middle cerebral artery; ML: machine-learning; MT: mechanical thrombectomy; NASCET: north american symptomatic carotid endarterectomy trial; NCCT: non-contrasted computed tomography; SVM: support-vector machines.

Table S2. Application of AI in cardioembolic source detection.

References	AI-based approaches	Data collection	Study aims and clinical implications	Results
Attia ZI et al. (2019)	CNNs	180 922 patients with 649 931 normal sinus rhytm ECGs for analysis: 456 789 ECGs from 126 526 patients in the training dataset, 64 340 ECGs from 18 116 patients in the validation dataset, 130 802 ECGs from 36 280 patients in the testing dataset	To develop a rapid, inexpensive point-of-care means of identifying patients with atrial fibrillation using ML	**Overall Accuracy** 83.3 % (83.0-83.7) **Sensitivity** 82.3 % (80.9-83.6) **Specificity** 83.4 % (83.0-83.8) **AUC** 0.90 (0.90-0.91)
Acharya UR et al. (2017)	CNNs	48-half-hour long ECG recording from 47 subjects, 109,449 ECG beats	To use a deep learning approach to identify the five different classes of abnormal ECG heartbeats	**Accuracy** 94.03% (noisy ECGs)

				93.47% (noise free ECGs)
Oh SL et al. (2019)	DL (modified U-net model)	48 ECG recordings from 47 subjects, all sampled at 360 Hz, derived from the MIT-BIH arrhythmia dataset from PhysioNet	To develop an automated computer aided diagnostic (CAD) system that can expedite the process of arrhythmia diagnosis	**Accuracy** 97.32%
Jeong HG et al. (2021)	DL (ASTRO-X deep neural network)	4064 chest radiographs of patients with acute ischaemic stroke (internal test), 750 chest radiographs of patients with acute ischemic stroke (external testing)	To elaborate a deep convolutional neural network that could diagnose cardioembolic stroke based on chest radiographs	**Accuracy** 84.4% (internal test) 74.1% (external test) **Sensitivity** 0.66 (internal test); 0.78 (external test) **Specificity** 0.92 (internal test); 0.73 (external test) **AUC** 0.86 (internal test); 0.82 (external test)
Awni YH et al. (2019)	DL	91,232 single-lead ECGs from 53,549 patients who used a single-lead ambulatory ECG monitoring device	To demonstrate that a deep learning approach (DNN) can classify a broad range of distinct arrhythmias from single-lead ECGs with high diagnostic performance similar to that of cardiologists (average cardiologist)	**AUC** 0.97 (DNN); 0.78 (average cardiologist)
Rocon C et al. (2020)	ML	108 patients with left ventricular non-compaction cardiomyopathy (LVNC)	To analyze a large set of echocardiographic (echo) and cardiac magnetic resonance imaging (CMRI) parameters using ML techniques to find imaging predictors of clinical outcomes in a long-term follow-up of LVNC patients	LVEF (CMRI), RV end-systolic volume (CMRI), RV systolic dysfunction (echo), and RV lower axis (CMRI): **Accuracy** 75.5% **Sensitivity** 77% **Specificity** 75%
Guan W et al. (2020)	ML	1598 patients with acute ischemic strokes	To assess the accuracy of automated algorithms to perform cardioembolic stroke subtyping using an electronic health record (EHR) database.	Random forest: **Accuracy** 92.2% **AUC** 91.1% (95% CI, 87.5%-93.9%)
Montanaro VVA et al. (2021)	AI	499 patients with CD and IS	To describe a multicenter cohort of patients with concomitant CD and IS to create a predictive model for cardioembolic embolism in CD and IS	Higher prevalence of vascular risk factors and lower median age in patients with cardioembolic etiology
Yang X et al. (2021)	CNNs	6500 12-lead ECG recordings (training set), 500 12-lead ECG recordings (testing set)	To propose a 12-lead ECG arrhythmia classification method using a cascaded convolutional neural network (CCNN) and expert features.	The method has been validated against the first China ECG Intelligence Challenge, obtaining

					a final score of 86.5% for classifying 12-lead ECG data with multiple labels into 9 categories	

AI: artificial intelligence; AUC: area under the curves; CNNs: convolutional neural networks; DL: deep-learning; ML: machine-learning; CAD: computer aided diagnostic; ASTRO-X: acute STROke classification by chest X-ray; DNN: deep neural network; LVNC: left ventricular non-compaction cardiomyopathy; CD:Chagas disease; IS: ischemic stroke; EHR: electronic health record; CCNN: cascaded convolutional neural network.

Table S3. Main Studies considering application of AI in Small Vessel Disease diagnosis.

References	Study aim	Date of pubblication	AI-based approaches	Main results	Clinical implications	Limitations
Lambert et al. [94]	Define patterns of grey matter alterations associated with SVD, and how they associated with WMH.	2015	Machine learning	Altered grey matter measures can predict WMH severity which is associated with cortical thinning.	WMH is associated with endothelial dysfunction and lacunar infarction. Being able to identify indirect signs of its severity could help the clinician to stratify cSVD patients hence their cerebrovascular risk.	Tissue segmentation system required a degree of manual correction. Use of multicollinearity in statistical analysis can lead to artificially inflated errors.
Ciulli et al. [95]	investigate the link between alterations in executive functions in patients with mild cognitive impairment and cSVD and to associate them to the brain substrates of this impairment	2016	Machine learning	Impaired performance in the TMT-B, can be predicted with high accuracy through an SVM-based machine learning strategy in MCI patients with SVD using DTI data alone.	Results of an easy to perform cognitive test could help to predict SVD risk and entity.	Small sample size
González-Castro et al. [99]	Develop and validate an automatic scheme to qualitatively classify T2-weighted MRI (as having none or few	2021	Deep Learning (CNN)	The deep learning system proposed had a good generalizability in the	Reduce analysis time	Limited testing of models. Limited MRI imaging modalities

	PVS compared with having many of them)			segmentation of WMH from different data sources		
Karel et al. [92]	identify potential novel biomarkers for cSVD	2022	Machine Learning	myeloperoxidase levels were altered	Implement SVD pathophysiology knowledge and hence implement diagnostic tools at our disposal	Small sample size. Low basic features homogeneity between case and control individuals.
Wang et al. [105]	assess the role of cerebral SVD in prediction models in patients with different subtypes of acute ischemic stroke (AIS).	2023	Machine Learning	Different SVD markers had distinct prognostic weights in AIS patients	Provide the clinician a prognostic tool to stratify patient risk	Use of qualitative rather than quantitative parameters . Small sample size. Narrow range of clinical variables.

SVD, small vessels disease; cSVD, cerebral small vessels disease; WMH, White Matter Hyperintensity, TMT- B, Trial Making test ; SVM, Support Vector Machines; MCI, Mild Cognitive Impairment; DTI, Diffusion Tensor Imaging .

Table S4. AI application in stroke of undetermined etiology.

**References**	**Study aim**	**Date of pubblication**	**AI-based approaches**	**Main results**	**Clinical implications**	**Limitations**
Kamel et al [111]	Detection of the probability of a cardioembolic source in a cohort of 580 patients with ESUS, using an automated algorithm able to distinguish cardio-embolic and non-cardioembolic strokes	2020.	ML	44% of strokes of undetermined source (ESUS) resulted from cardioembolism. The model was able to identify a cardioembolic source with high accuracy (AUC 0.85, 95% credibility interval) associated with the eventual detection of AF (OR per 10% increase in predicted probability of cardioembolic	Identifying population at higher risk of occult cardioembolism (older patients,cardiac disease, reduced left ventricular sistolic function, left atrial enlargement) to be treated with anticoagulant therapy.	Data from a single academic center; unavailability of comparison with a gold standard; lack of pixel-level brain imaging for improving performance.

				source, 1.27; 95% CI, 1.03–1.57)		
Ntaios et al. [112]	Identification of potential embolic sources in ESUS and the risk of stroke recurrence	2021	ML	The study identified four clusters of ESUS with similar risk of stroke recurrence: arterial disease (50%), PFO (15-20%), atrial cardiopathy (15-20%) and left ventricular disease (5%). AF was not associated with any cluster.	The acknowledgement of the pathological mechanism of ESUS (arterial disease vs cardioembolic source) may guide the choice for the better therapeutic strategy (anticoagulant vs aspirin) to prevent recurrent ischemic cerebrovascular events	Limited size of the sample; the use of an empirical method based on clustering algorithms; risk of bias due to bugs in the registration and the workup of the patients during the in-hospital phase.
Luo et al. [113]	Identification of predictive features associated with higher risk of adverse outcome in post-closure PFO patients	2022	ML	Among 197 patients diagnosed with PFO-associated ESUS underwent to closure treatment, patients with some clinical features (male gender, higher systolic and diastolic blood pressure, higher BMI, lower HDLC and increased proportion of presence of atrial septal aneurism, etc.) were at higher risk of stroke recurrence	The automated stratification risk of stroke recurrence in patients with PFO may guide the best therapeutic choice, identifying the population which may benefit the most from the foramen closure	Lack of pre- and post-procedural information (post-hoc analysis); small size of the study cohort; need of validation of the model by external datasets; misdiagnosis of occult AF
Esenwa et al. [114]	Detection of inflammation and coagulation biomarkers defining a new subtype of ischemic stroke associated	2021	ML	The study identifyed 4 unique clusters on the basis of COVID-19 infection severity: patients with higher COVID-19 disease severity (defined as	The model allows to estimate the risk of cerebrovascular events in COVID-19 patients	Selection of only imaging confirmed cases qith underestimation of AIS and thrombotic events; limited availability of biomarkers;

with COVID19 infection	mortality) and coagulopathy (defined by elevated D-dimer levels) had higher prevalence of AIS with a strong association with cryptogenic cause, while no ischemic events were reported in patients with mild disease.	wide confidence intervals in the definition of the accuracy with D-dimer levels associationwith stroke

AF: atrial fibrillation ; AIS: acute ischemic stroke; AUC: area under the curve; BMI: body mass index; CI: confidence interval; COVID-19: coronavirus disease 2019ESUS: embolic stroke of an undetermined source; HDLC: high-density lipoprotein cholesterol; ML: machine learning; OR: Odds ratio; PFO: patent foramen ovale.
